# Supplementary material for: Glycolytic flux control by drugging phosphoglycolate phosphatase
Source: Nat Commun. 2022 Nov 11;13:6845. doi: 10.1038/s41467-022-34228-2 (PMC9652372; doi:10.1038/s41467-022-34228-2)
Supplement: Supplementary file 1 — Supplementary Info File #1 [file 41467_2022_34228_MOESM1_ESM.pdf]

Glycolytic flux control by drugging phosphoglycolate phosphatase

SUPPLEMENTARY INFORMATION

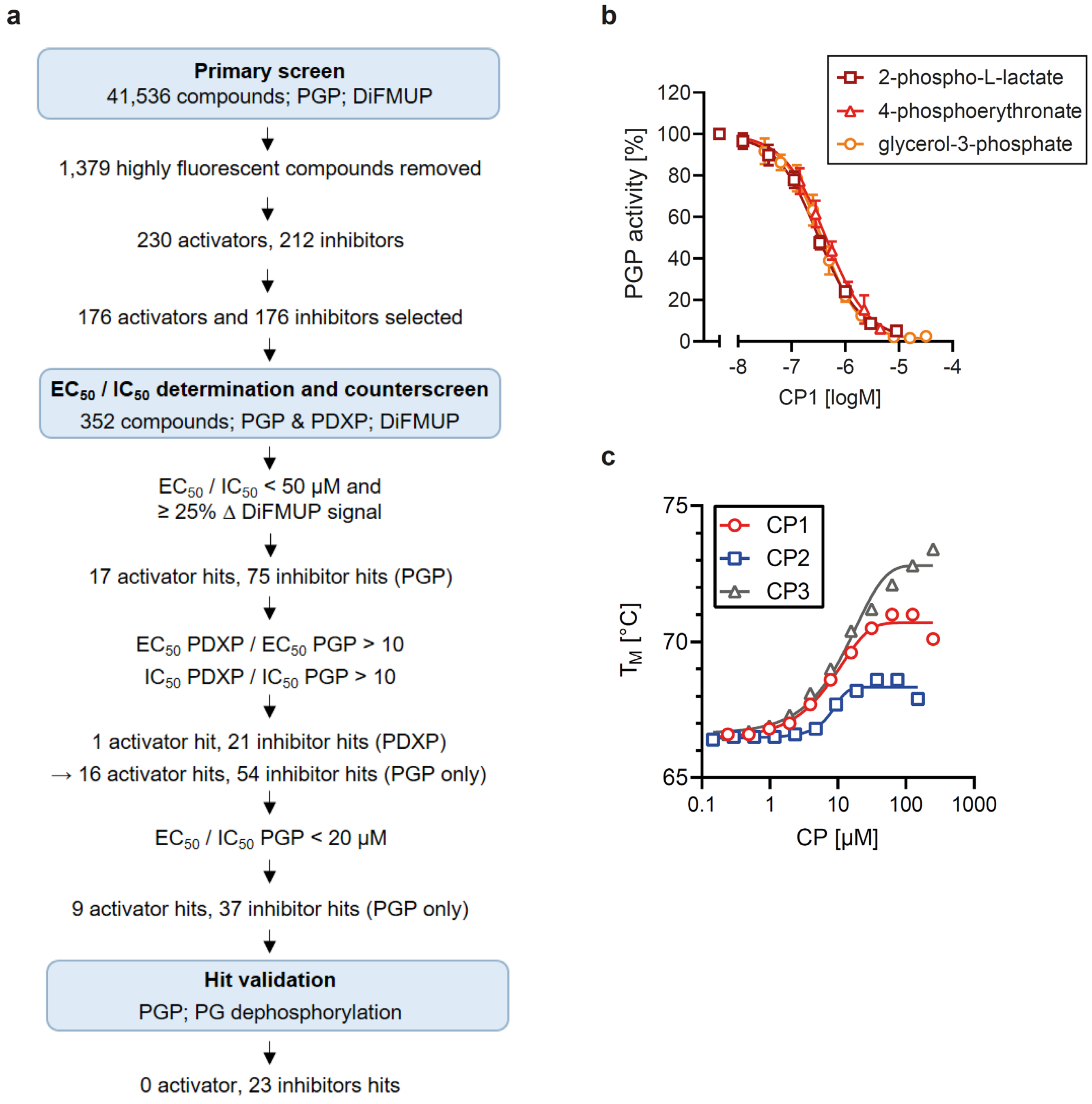

Supplementary Figure 1 (related to Figure 1): Identification and characterization of PGP inhibitors. (a) Schematic of the screening campaign for small molecule PGP modulators. A

primary screen was conducted using 6,8-difluoro-4-methylumbelliferyl phosphate (DiFMUP) as an artificial substrate. Out of 41,536 screened compounds, 1379 compounds were discarded that showed very high autofluorescence (as recognized by elevated fluorescence at the start of the kinetic curve), 230 compounds showed statistically significant PGP activation, and 212 compounds showed PGP inhibition (as recognized by an elevated or decreased slope of the kinetic curve). The average Z' factor of the screen was 0.62 ± 0.12. The 176 most active compounds of both groups (activators and inhibitors) were then selected for DiFMUP-based concentration-dependent validation. A counter-screening was conducted with these samples in parallel, also in a concentration-dependent fashion, against the PGP paralog and closest relative, pyridoxal phosphatase (PDXP). The compounds that were inactive against PDXP were validated in a secondary assay, using the PGP substrate 2-phosphoglycolate (PG). None of the nine PGP activator hits increased PGP activity towards PG, suggesting false positive results, presumably due to the fluorescent DiFMUP readout. In contrast, 23 of the 37 PGP inhibitor hits blocked PG dephosphorylation by >50%, and IC_{50} values were determined for these 23 compounds using PG as a substrate (see Supplementary Table 1). (b) Determination of half-maximal inhibitory constants (IC_{50}) of compound **1** (CP1), using purified murine PGP and 2-phospho-L-lactate, 4-phosphoerythronate or glycerol-3-phosphate as substrates. Error bars not shown are hidden by the symbols. Data are mean values ± S.D. of *n*=3 independent experiments. (c) Differential scanning fluorimetry analyses with purified murine PGP in the presence of serial dilutions of CP1-3. The shift in melting temperature (ΔT_M) upon CP-binding was monitored with Sypro Orange. Shown are normalized, averaged and fitted data from triplicate determinations (*n*=1). Source data are available as Source Data File.

a

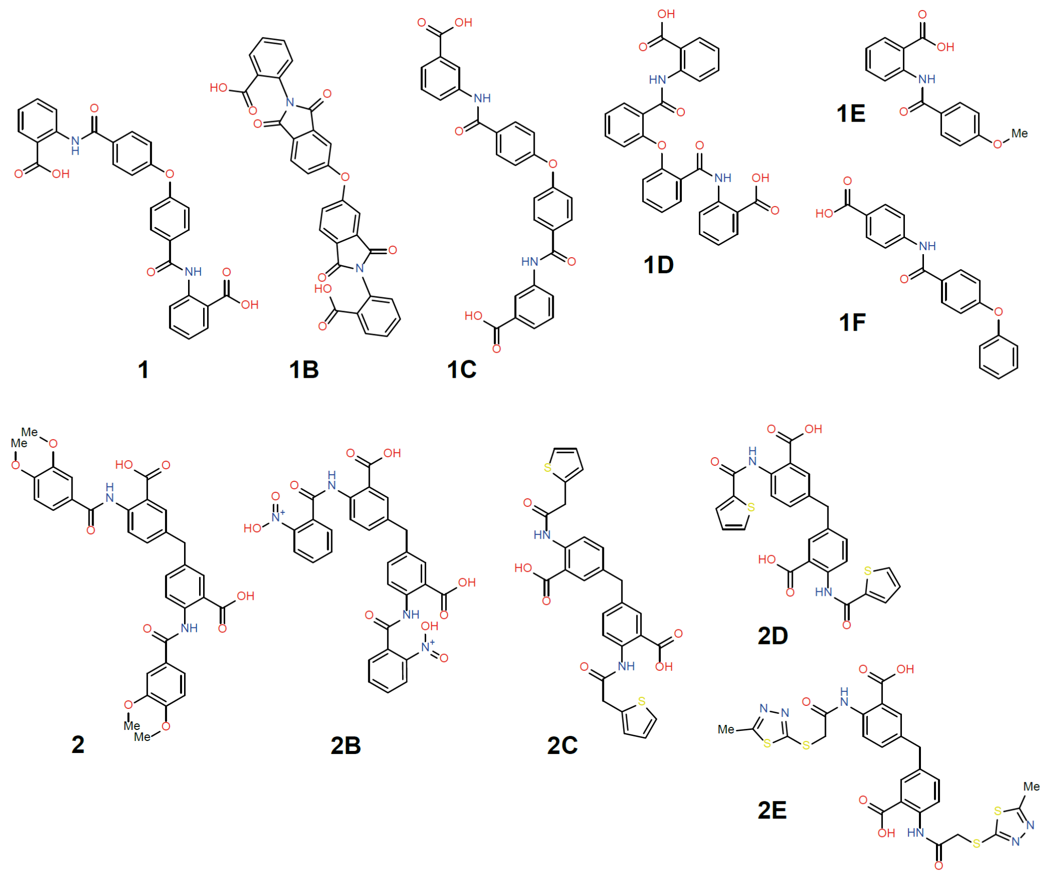

b

CP	IC_{50} (μM)	Inhibition (%)
**1**	0.38 ± 0.08	97.42 ± 2.24
**1B**	n.d.	24.80 ± 2.45
**1C**	27.78 ± 6.29	62.94 ± 2.99
**1D**	n.d.	21.07 ± 3.76
**1E**	n.d.	38.82 ± 3.49
**1F**	n.d.	11.94 ± 3.47
**2**	1.62 ± 0.48	90.26 ± 0.58
**2B**	1.07 ± 0.01	99.08 ± 0.83
**2C**	15.96 ± 2.34	79.93 ± 0.30
**2D**	1.71 ± 0.03	92.92 ± 0.62
**2E**	n.d.	39.20 ± 2.15

c

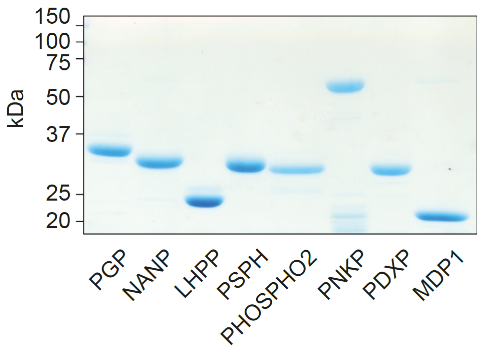

d

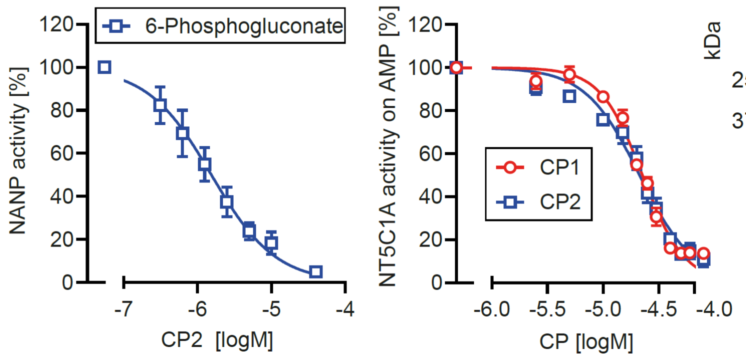

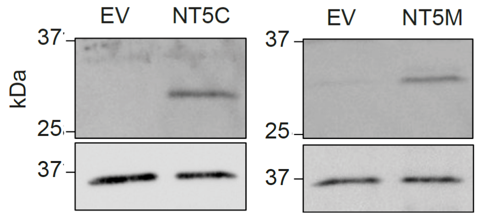

Supplementary Figure 2 (related to Figure 1): Characterization of CP1 and CP2 analogs. (a) 2D structures of CP1, CP2, and their analogs. (b) Effects of CP1, CP2, and their analogs on PGP phosphatase activity were assessed using recombinant, purified murine PGP and PG as a substrate. Compounds were initially tested at a concentration of 40 µM, and IC_{50} values were determined if the compound inhibited PGP activity by more than 50% compared to the DMSO solvent control. All results are mean values ± S.D. of *n*=3 independent experiments; n.d., not determined. (c) Upper panel, purity of the tested, recombinant phosphatases (10 µg of protein/lane). A Coomassie Blue-stained gel is shown. Tested enzymes that are not included in this panel were obtained commercially. Lower panel, Western blots showing the overexpression of the myc-tagged cytosolic 5', 3'-nucleotidase NT5C and mitochondrial 5', 3'-nucleotidase NT5M in HEK293 cells (uncropped blots in Source Data). (d) Determination of half-maximal inhibitory constants (IC_{50}) for CP2 inhibition of NANP activity on 6-phosphogluconate (left panel), and CP1 or CP2 inhibition of NT5C1A activity on AMP (right panel). Error bars not shown are hidden by the symbols. Data are mean values ± S.E.M. of *n*=3 independent experiments. Source data are available as Source Data File.

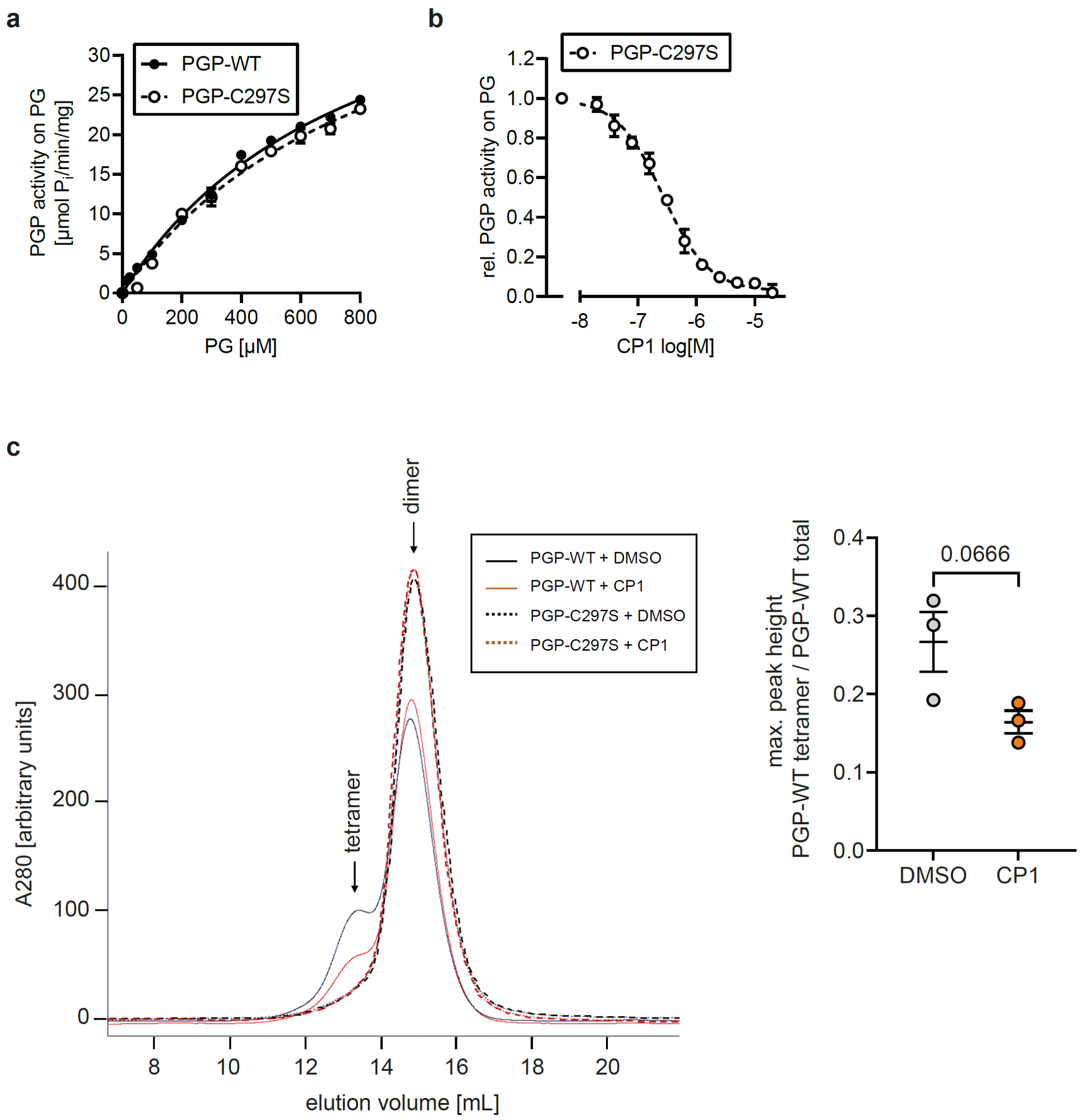

Supplementary Figure 3 (related to Figure 2): Characterization of the homodimeric PGP variant PGP^{C297S}. (a) In vitro phosphatase activity of purified murine PGP^{C297S} in comparison to purified murine PGP^{WT}, using 2-phosphoglycolate (PG) as substrate. (b) Determination of the IC_{50} values of CP1 for PGP^{C297S}. Purified PGP^{C297S} was incubated with serial dilutions of CP1. PG dephosphorylation was quantified using malachite green. All data in (a) and (b) are mean values ± S.E.M of n=3 independent experiments. Error bars not shown are hidden by the symbols. (c) PGP self-association in the absence or presence of CP1. Size-exclusion chromatography (SEC) of purified, recombinant murine PGP-WT in comparison to PGP-C297

(30 µM each) was performed in the absence (0.1% DMSO solvent control) or presence of CP1 (100 µM). SEC profiles of PGP-WT are the mean of *n*=3 independent experiments; PGP-C297S ± CP1 was profiled once for comparison (*n*=1). The right panel shows a quantification of the effect of CP1 on PGP-WT tetramer peak heights; data are mean values ± S.E.M. of *n*=3 independent experiments. Statistical testing was done using a two-sided *t*-test, and the *p*-value is indicated. Source data are available as Source Data File.

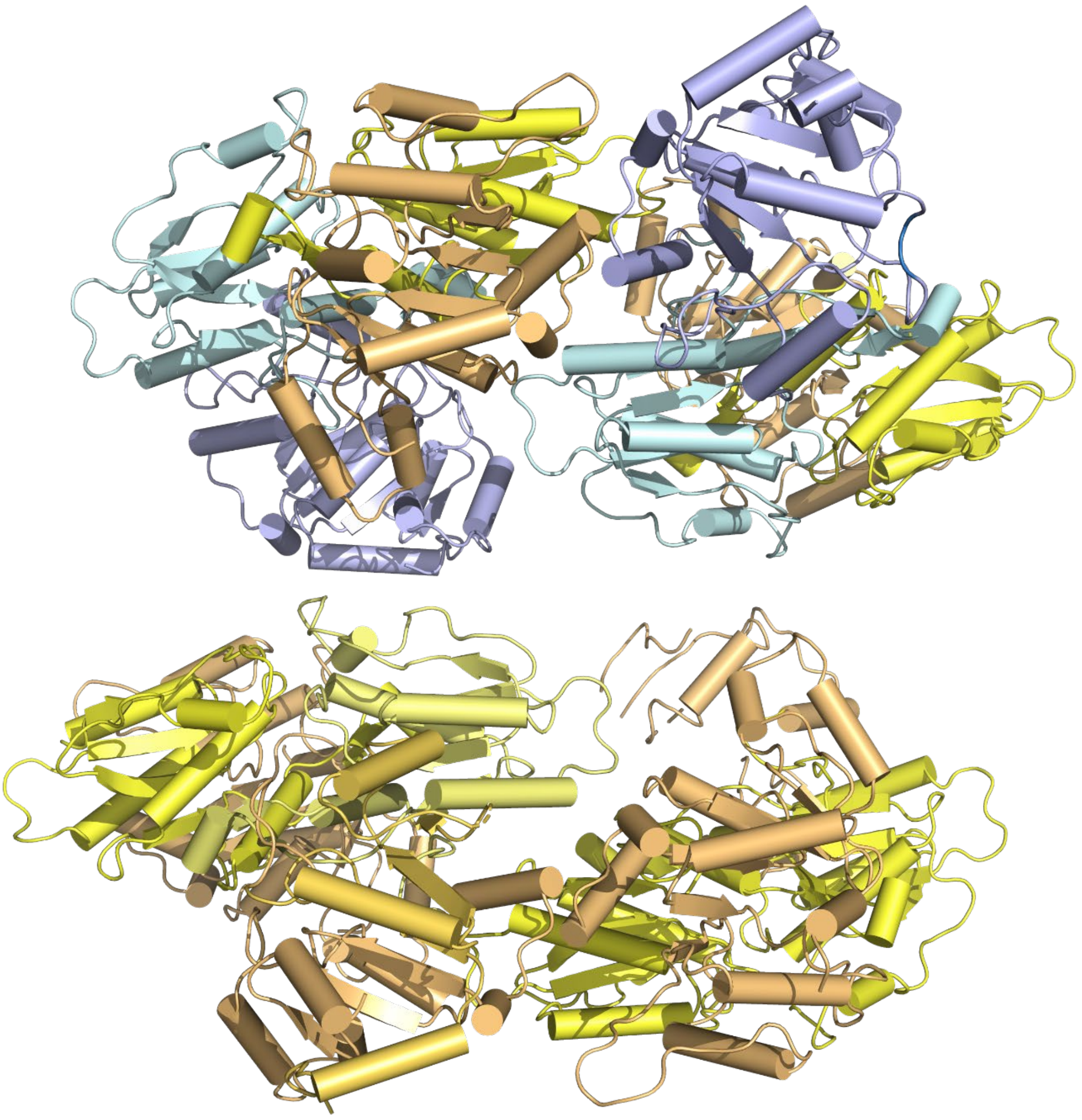

Supplementary Figure 4 (related to Figure 2): Arrangement of the four dimers in the P1 unit cell of apo-PGP. The view is along a non-crystallographic two-fold symmetry axis. Chain D and chain E monomers (crystallized in the cap open conformation) are located on the same side (on top in this representation) and hence do not follow the non-crystallographic symmetry.

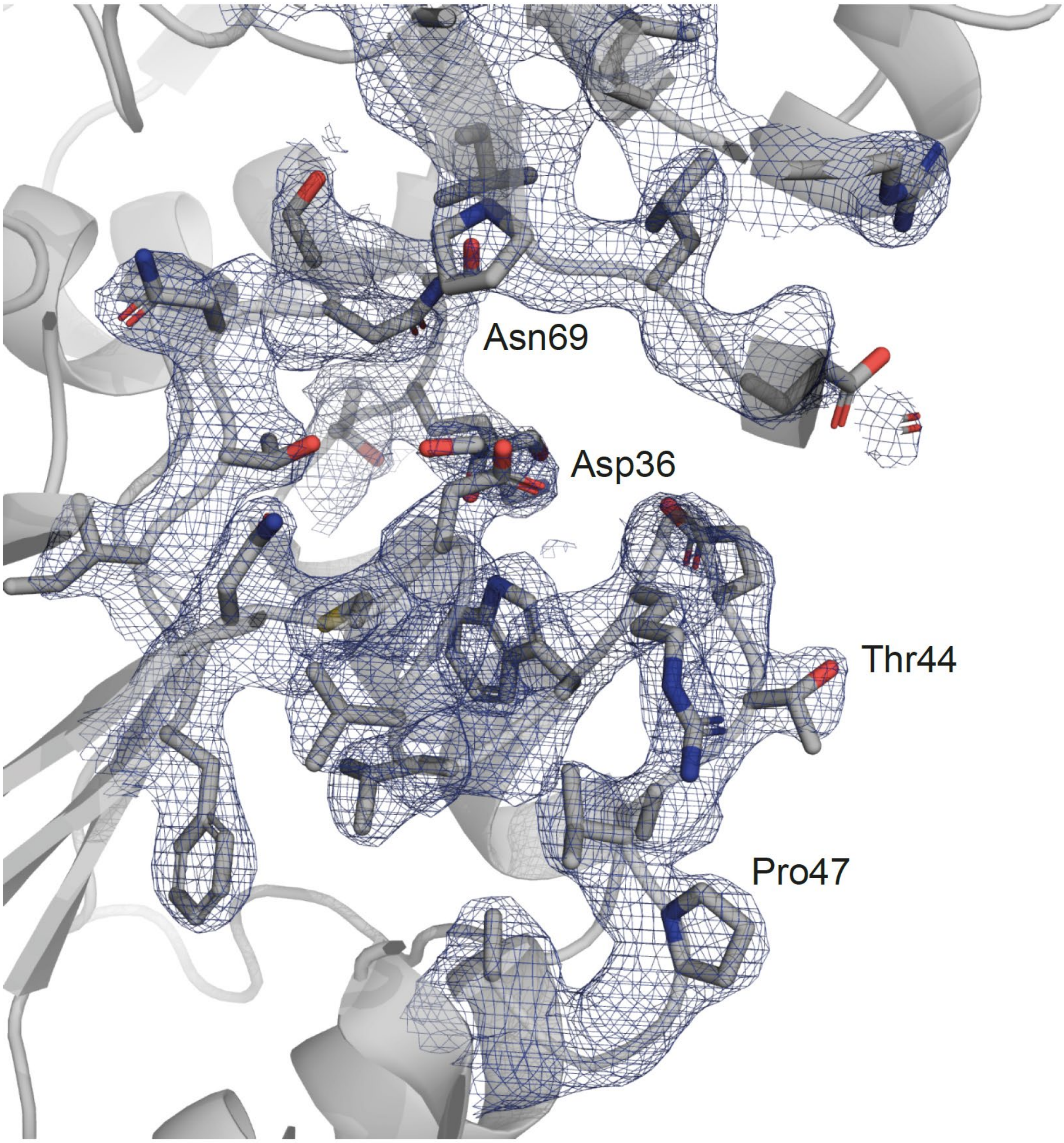

Supplementary Figure 5 (related to Figure 2): Electron density map of the active site of apo-PGP (monomer E). A sigma-A-weighted $2Fo - Fc$ map contoured at an RMSD of 1.0 is shown.

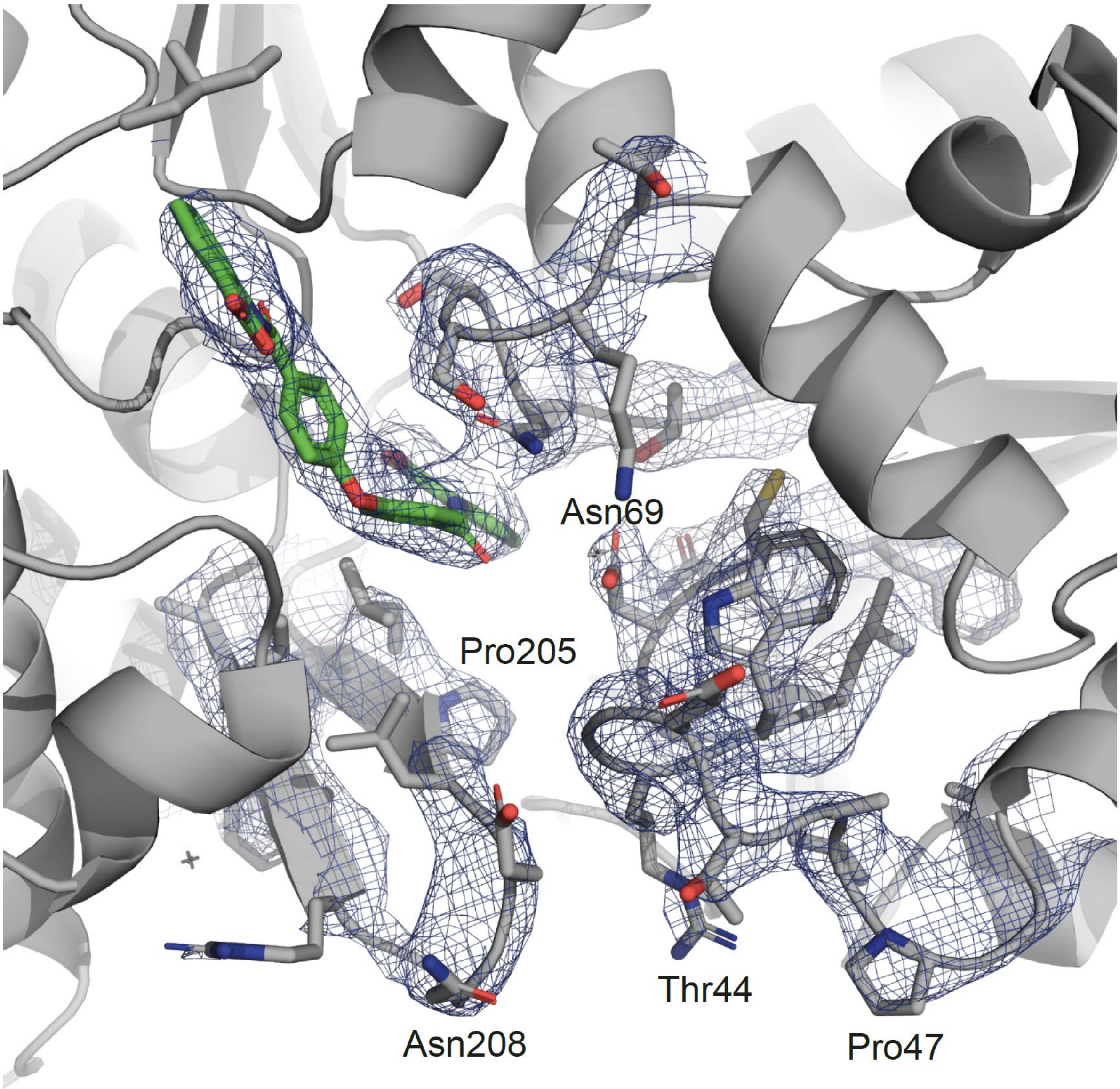

Supplementary Figure 6 (related to Figure 3): Electron density map of the active site of CP1-bound PGP (monomer A). A sigma-A-weighted $2Fo - Fc$ map contoured at an RMSD of 1.0 is shown.

```
PGP_MOUSE MAEAEAGGDEARCVRLSAERAKLLLAEVDTLLFDCDGVLWRGETAVPGAPETLRALRARG 60
PGP_HUMAN MAAAEAGGDDARCVRLSAERAQALLADVDTLLFDCDGVLWRGETAVPGAPEALRALRARG 60
          ** ******:***********: ***:************************:********

PGP_MOUSE KRLGFITNNSSKTRTAYAEKLRRLGFGGPVGPEAGLEVFGTAYCSALYLRQRLAGVPDPK 120
PGP_HUMAN KRLGFITNNSSKTRAAYAEKLRRLGFGGPAGPGASLEVFGTAYCTALYLRQRLAGAPAPK 120
          **************:**************.** *.*********:**********.* **

PGP_MOUSE AYVLGSPALAAELEAVGVTSVGVGPDVLHGDGPSDWLAVPLEPDVRAVVVGFDPHFSYMK 180
PGP_HUMAN AYVLGSPALAAELEAVGVASVGVGPEPLQGEGPGDWLHAPLEPDVRAVVVGFDPHFSYMK 180
          ******************:******: *:*:**.*** .*********************

PGP_MOUSE LTKAVRYLQQPDCLLVGTNMDNRLPLENGRFIAGTGCLVRAVEMAAQRQADIIGKPSRFI 240
PGP_HUMAN LTKALRYLQQPGCLLVGTNMDNRLPLENGRFIAGTGCLVRAVEMAAQRQADIIGKPSRFI 240
          ****:******.************************************************

PGP_MOUSE FDCVSQEYGINPERTVMVGDRLDTDILLGSTCSLKTILTLTGVSSLEDVKSNQESDCMFK 300
PGP_HUMAN FDCVSQEYGINPERTVMVGDRLDTDILLGATCGLKTILTLTGVSTLGDVKNNQESDCVSK 300
          *****************************:**.***********:* ***.******: *

PGP_MOUSE KKMVPDFYVDSIADLLPALQG 321
PGP_HUMAN KKMVPDFYVDSIADLLPALQG 321
          *********************
```

Supplementary Figure 7 (related to Figures 3 and 4). Protein sequences of murine PGP (UniProtKB Q8CHP8) and human PGP (UniProtKB A6NDG6) were aligned with the EMBL-EBI multiple sequence alignment tool Clustal Omega version 1.2.4. PGP residues found to engage in CP1 interactions (red color) are identical in murine and human PGP.

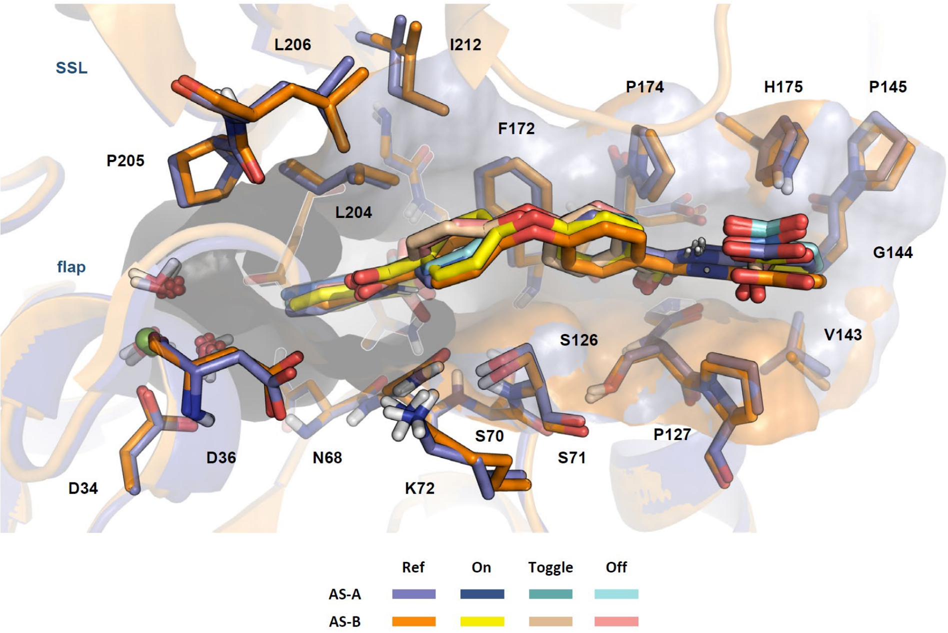

	Water mode	DSX rescoring Total	DSX rescoring PAS	RMSD (Å)	Poses in cluster
AS-A	On	-146.41	-3.96	0.48	192
	Toggle	-146.96	-3.97	0.44	187
	Off	-144.44	-3.90	0.50	197
AS-B	On	-151.19	-4.09	0.73	200
	Toggle	-156.92	-4.24	1.04	200
	Off	-153.66	-4.15	1.05	200

Supplementary Figure 8. Top-ranked binding modes for CP1 obtained after rescoring with DSX. For each of the two active sites (AS-A and AS-B), docking results for all three water modes are shown in the table, along with the total DSX score and the DSX per-atom-score (PAS), the RMSD with respect to the crystallographic binding mode of CP1 in PGP$^{D34N;C297S}$ and the number of docking poses belonging to the same cluster as the top-ranked pose. The figure illustrates these binding modes after superpositioning AS-B (orange) onto AS-A (blue) based on the core residues. The catalytically essential Mg^{2+} ion is shown as a green sphere, along with the coordinated water molecules. Neither water replacement nor direct coordination to the Mg^{2+} ion was observed in any of the docking solutions. Instead, the docking runs showed nearly perfect convergence to the experimentally observed binding mode of CP1 in PGP$^{D34N;C297S}$. Source data are available as Source Data File.

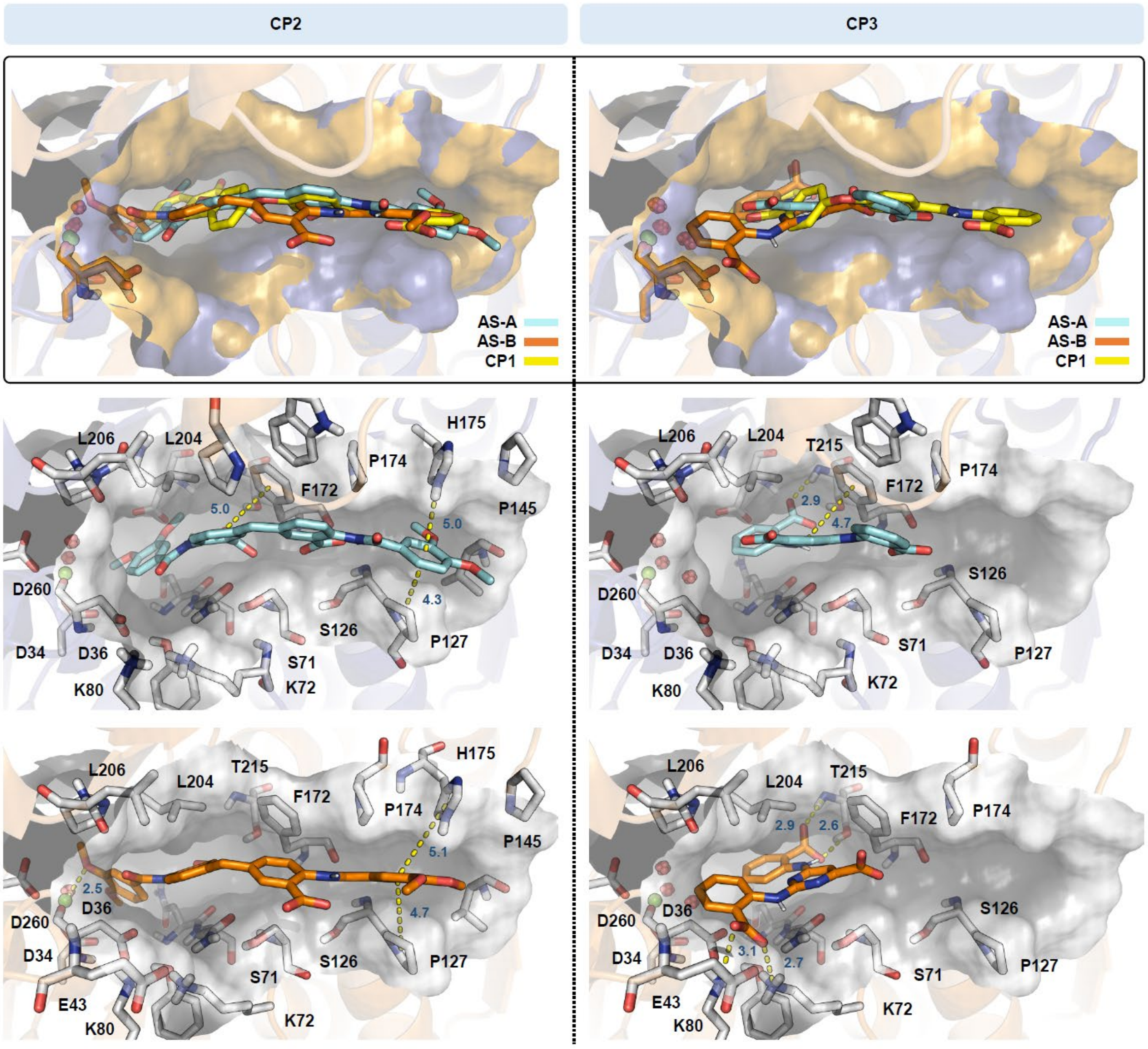

	DSX rescoring Total	PAS	Rank	Poses in cluster	Water molecules
CP2	-156.35	-3.47	1 AS-A	2	2
	-156.98	-3.49	1 AS-B	34	1
CP3	-113.75	-3.92	1 AS-A	112	3
	-125.68	-4.33	1 AS-B	12	3

Supplementary Figure 9. Top-ranked binding modes for CP2 and CP3 in each of the two active sites of PGP obtained after rescoring with DSX. The three water molecules coordinated to the Mg^{2+} ion were considered in toggle mode; the number of preserved water molecules in the corresponding docking solution is reported in the last column of the table. In the upper panel of the figure, the results in the two active sites are superimposed and compared with the crystallographic binding mode of CP1. The lower panels show the individual docking poses, with distances given in Å. The results indicate that well-scoring binding modes largely

overlapping with CP1 are feasible. However, in contrast to CP1, no such clear convergence to a single preferred binding orientation was obtained. Source data are available as Source Data File.

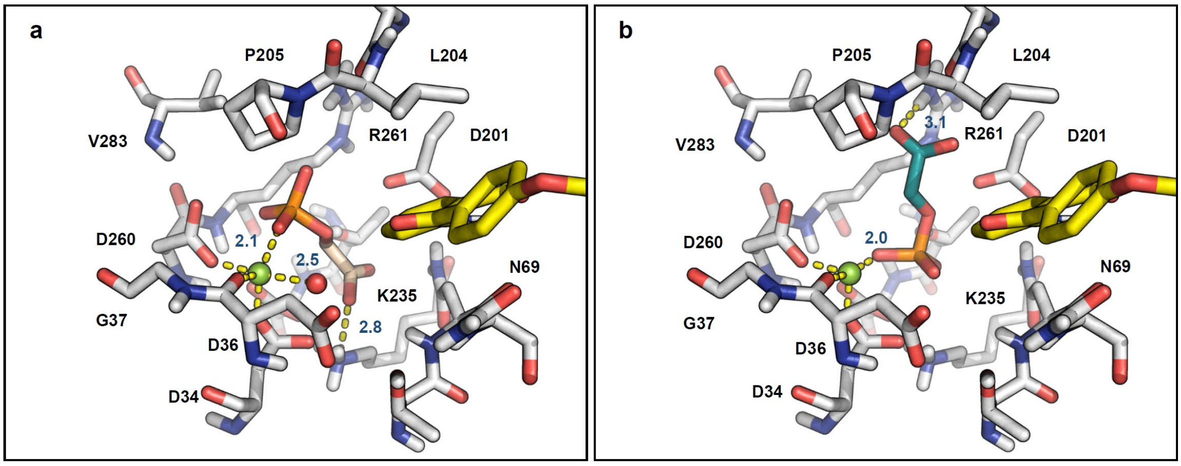

	Water molecules		DSX rescoring			Poses in
	Mode	Preserved	Total	PAS	Rank	cluster
a	toggle	1	-35.96	-3.99	1	50
b	off	-	-33.01	-3.67	1	51

Supplementary Figure 10. Top-ranked binding modes after rescoring with DSX of 2-PG docked in two different water modes to the PGP active site of monomer B (AS-B) in the presence of CP1. Distances are given in Å, the reference structure CP1 is depicted in yellow sticks. The results indicate two possible binding modes of 2-PG, which are both non-overlapping with the CP1 binding site, but differ in the coordination of the phosphate group to the Mg^{2+} ion and in the orientation of the carboxylate, forming a hydrogen bond either with Lys235 (a) or with Arg261 in the opposite direction (b). Source data are available as Source Data File.

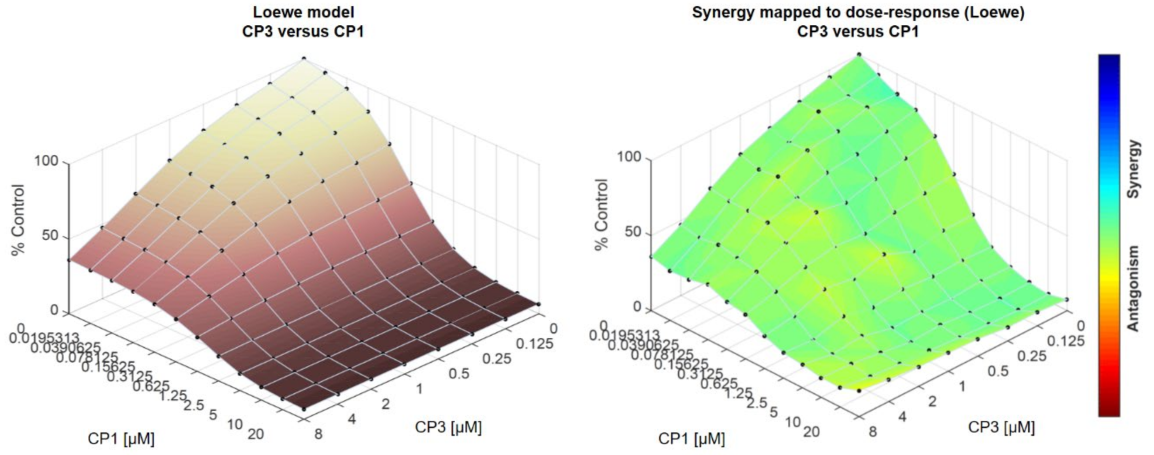

Supplementary Figure 11: CP1 and CP3 combination assays. PGP phosphatase activity measurements were conducted in the simultaneous presence of CP1 and CP3, using recombinant, purified murine PGP and PG as substrate. All wells contained 0.1% DMSO as a solvent control; control wells contained 0.1% DMSO only. Results are mean values ± S.E.M. of *n*=3 biologically independent experiments**.** Data were processed with Combenefit 2.021 software using the classical Loewe synergy model. Left panel, model-generated reference dose-response (prediction of effect if drugs are not synergistic), based on single agent dose-response curves. Left panel, resulting synergy distribution in the CP1/CP3 concentration space. Source data are available as Source Data File.

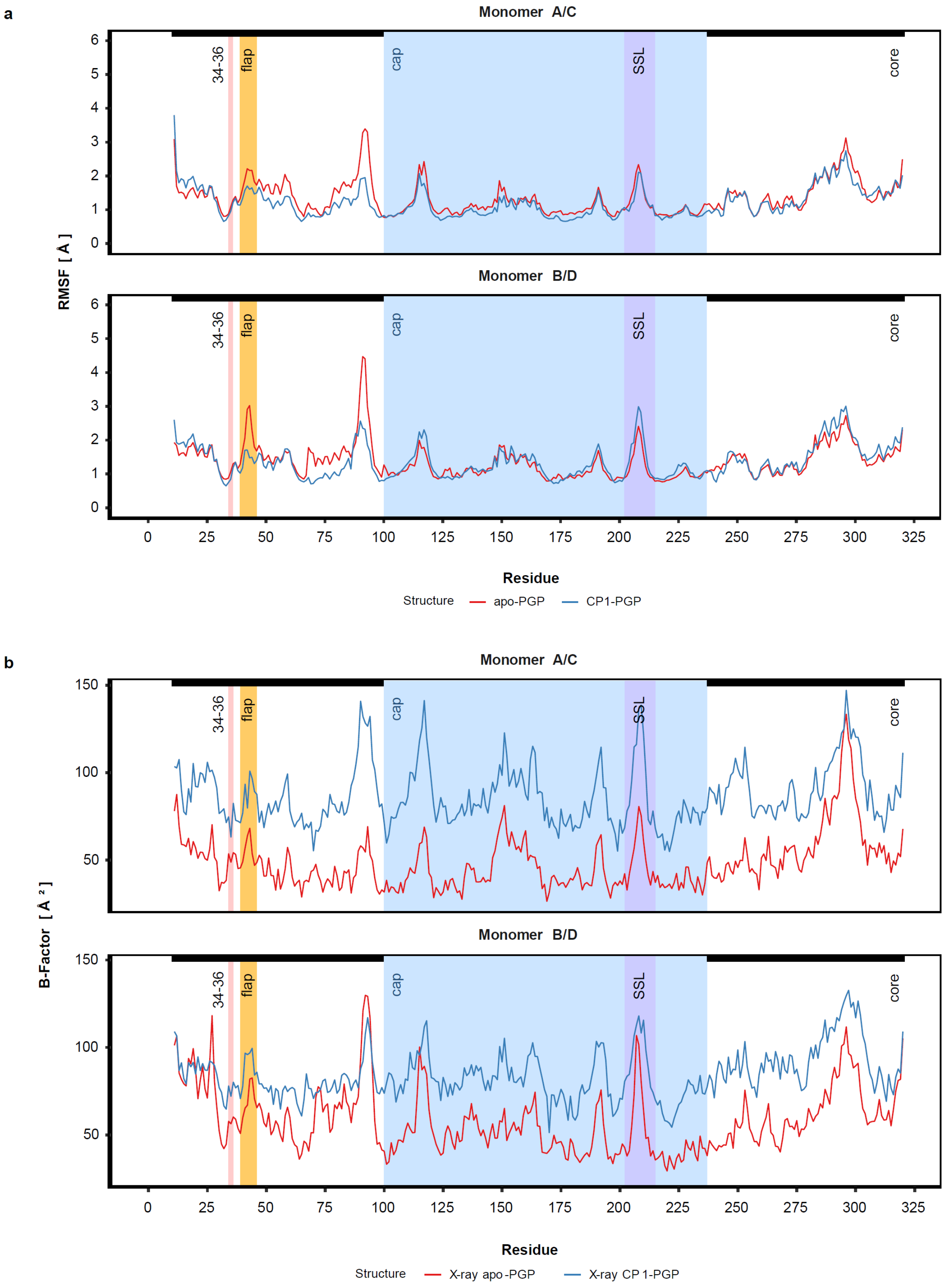

Supplementary Figure 12. RMS fluctuations from MD simulations and crystallographic temperature factors (B-values). (a) Root mean square fluctuations (RMSF) of the C$^{\alpha}$ atoms

calculated from the MD trajectories of apo-PGP (red line) and CP1-PGP (blue line). The blue area contains the cap residues, whereas the black bar indicates the core residues. The catalytic site (34-36), flap and substrate specificity loop (SSL) are further highlighted. Fluctuations in the flap and the subsequent regions of the core domain are larger for apo-PGP than for CP1-bound PGP. (b) Crystallographic temperature factors (B-values) of the C^{α} atoms in the crystal structures of apo-PGP (red line) and CP1-PGP (blue line), shown for comparison with the RMSF profile from the MD simulations. Absolute values between the different crystal structures should not be compared, only relative differences within a given structure are relevant. Source data are available as Source Data File.

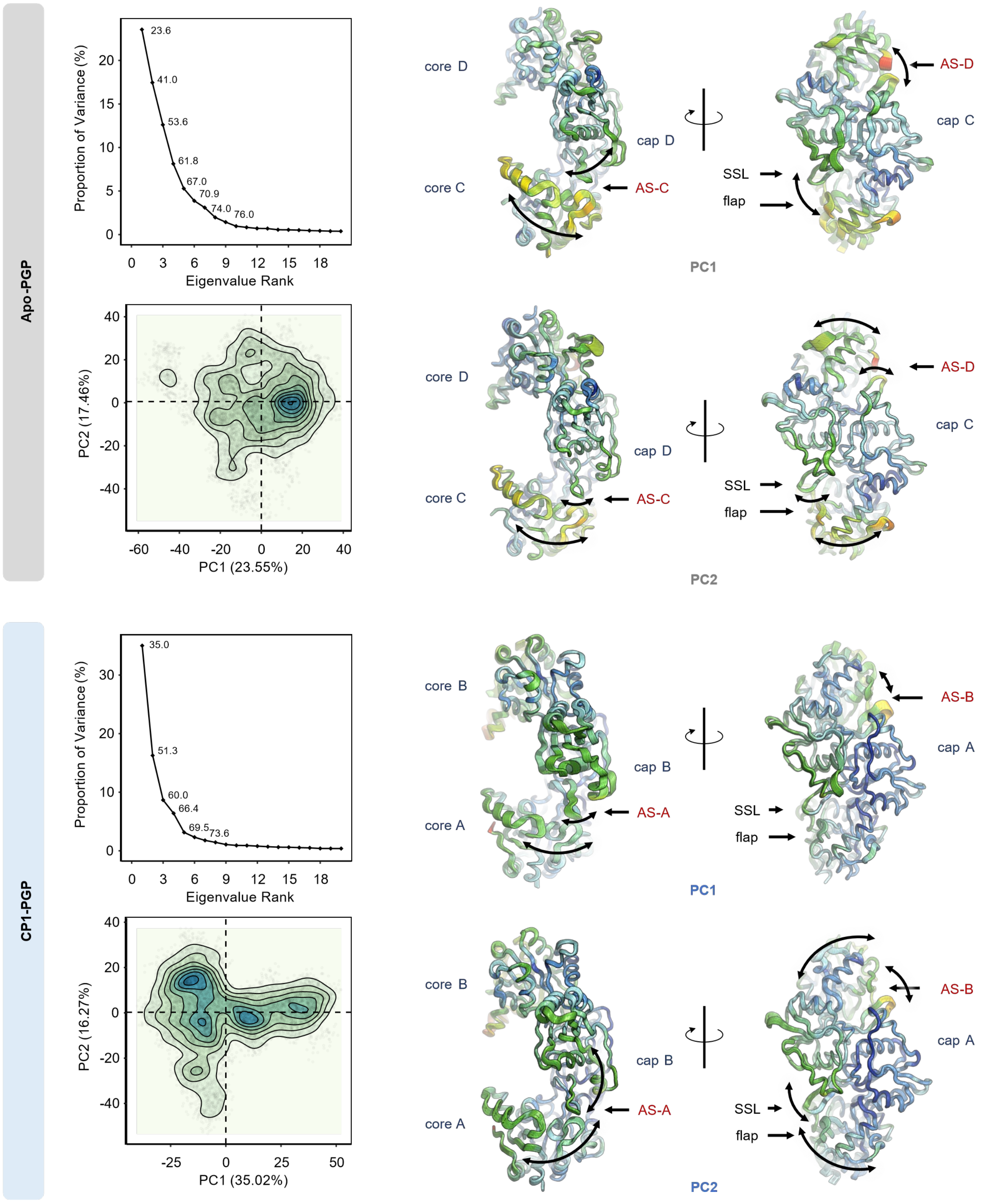

Supplementary Figure 13. Principal component analysis of the MD simulations. PCA plots and projections for the apo-PGP (top panel) and CP1-PGP MD simulations (bottom panel). The first plot (scree plot at the top left of each panel) shows the percentage of total variance (mean square displacement) captured by the first 20 principal components (PCs). The second diagram

(bottom left in each panel) shows density plots for the projections of all 10,000 MD trajectory frames (saved at 10 ps intervals) onto the subspace spanned by the first two PCs. The figures at the right illustrate the first two PCs for each simulation by means of an interpolation between the most dissimilar structures along the corresponding PC, colored according to the magnitude of the atomic displacement from blue (low) to orange/red (high). Movements along these PCs are furthermore visualized in the Supplementary Movies 1-4. They illustrate relative motions of the monomers with respect to each other, as a well as a slight clamshell-like opening-closing vibration of the core domains in case of CP1-PGP. Source data are available as Source Data File.

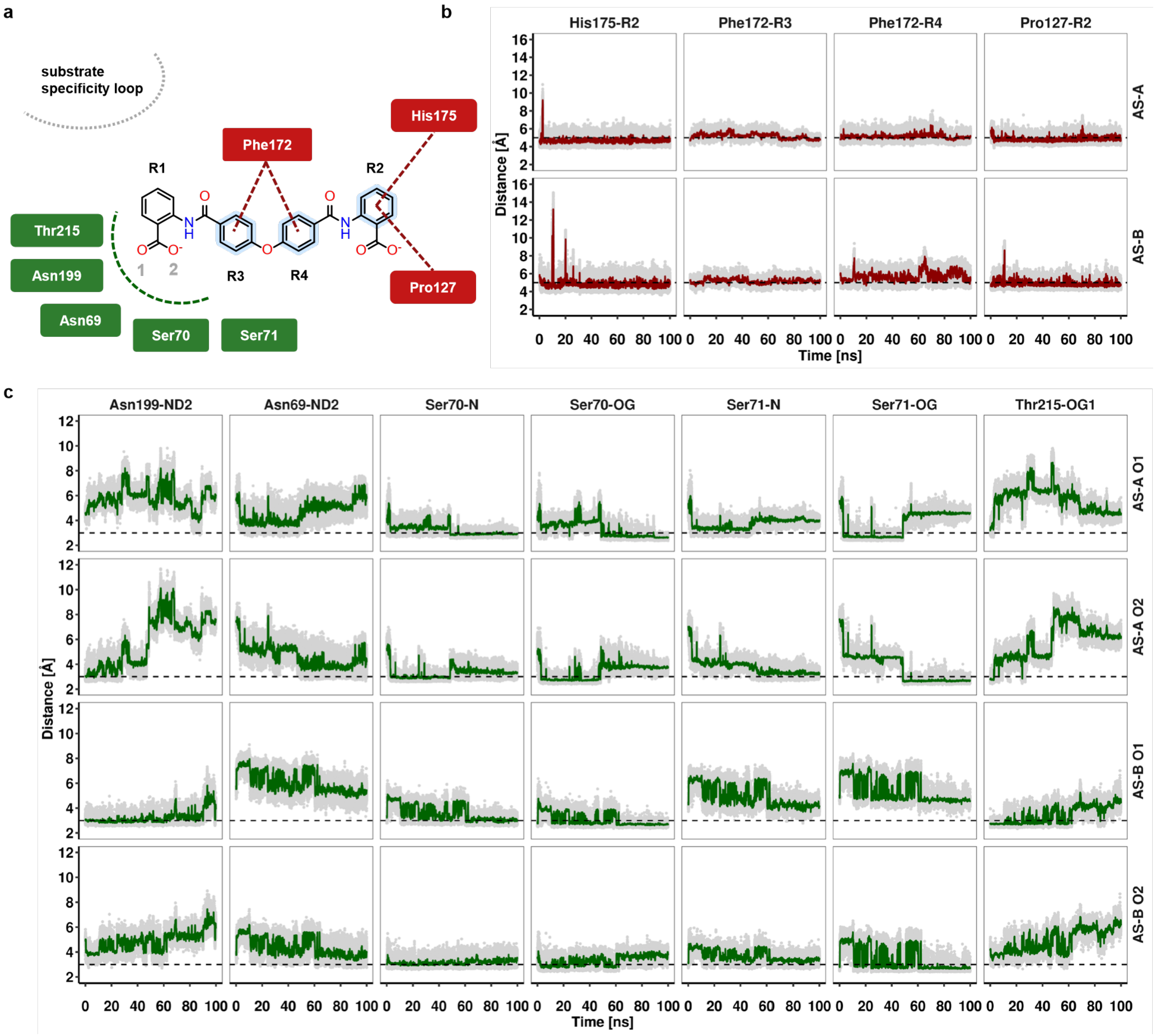

Supplementary Figure 14 (related to Figure 4). Distance analysis of CP1-PGP interactions in the MD simulations. (a) Schematic of PGP-residues and CP1 atoms involved in hydrogen bonds, aromatic or van der Waals interactions. Oxygen atoms (O1 and O2) from the deeply buried benzoate at the active site were considered for hydrogen bond formation with Thr215, Asn199, Asn69, Ser70, and Ser71 (green). Further distances were measured between the centers of mass of the CP1 phenyl rings R2-4 and the ring systems of Pro127, Phe172, and His175 (red). Timelines of the distances along the 100 ns trajectory are shown in (b) and (c) for CP1 in both active sites (AS-A and AS-B). A black dashed line is shown at 5 Å for van der Waals interactions (b) and at 3 Å for hydrogen-bond interactions (c), respectively. Source data are available as Source Data File.

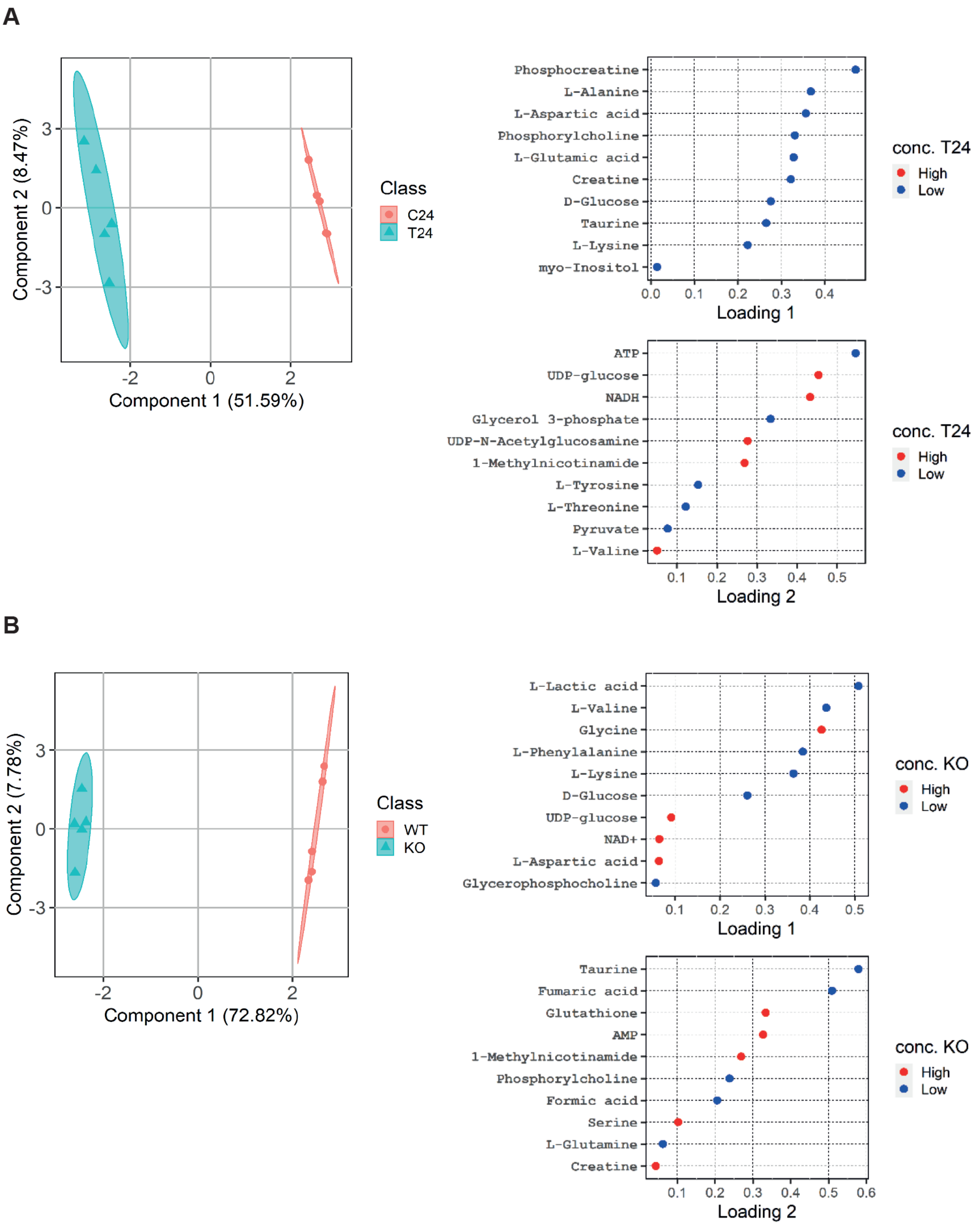

Supplementary Figure 15 (related to Figure 5): Classification of NMR metabolomic data by discriminant analysis. sPLS-DA results obtained for the comparison of (A) samples treated with the DMSO solvent control or 100 µM CP1 for 24 h (C24 and T24) and (B) of PGP-KO (clone C2) and PGP-WT (clone D5) cell lines. Left panels, 2D scores plot. Right panels, loading plots showing the composition of the principal components depicted in the scores plot. Metabolites labeled as high or low displayed a larger or smaller group mean value, respectively,

than the corresponding compared group. Source data are available as Source Data File, and raw datasets are available [1].

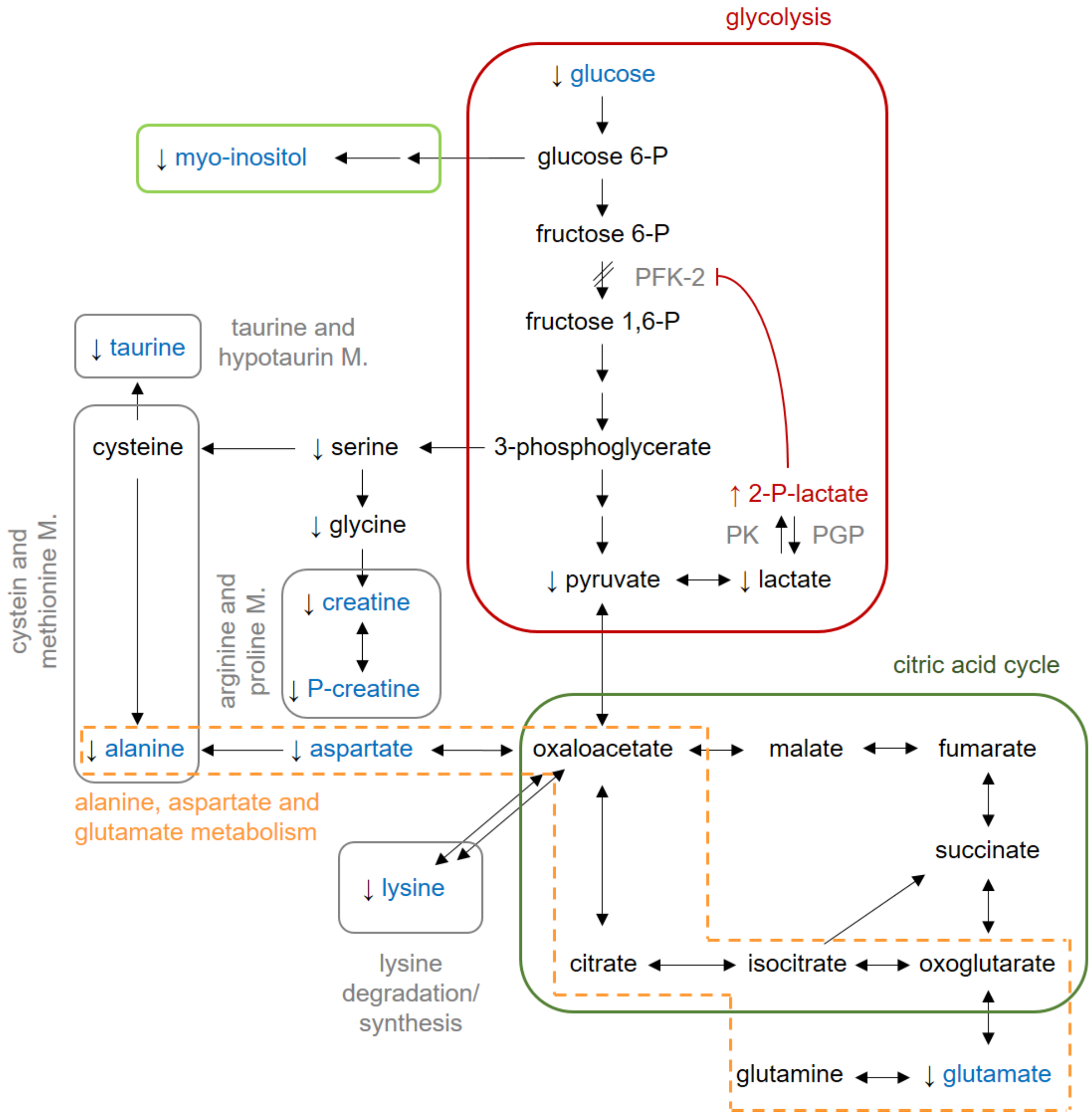

Supplementary Figure 16 (related to Supplementary Figure 15): Flowchart illustrating possible influences of PGP inhibition on metabolic pathways (colored frames). Nine of the ten determinative metabolites identified in Supplementary Figure 12 are shown in this flowchart (blue color). PGP, phosphoglycolate phosphatase; PK, pyruvate kinase; PFK-2, phosphofructokinase-2; M., metabolism; P, phosphate. ↑ Metabolite increased; ↓ metabolite decreased.

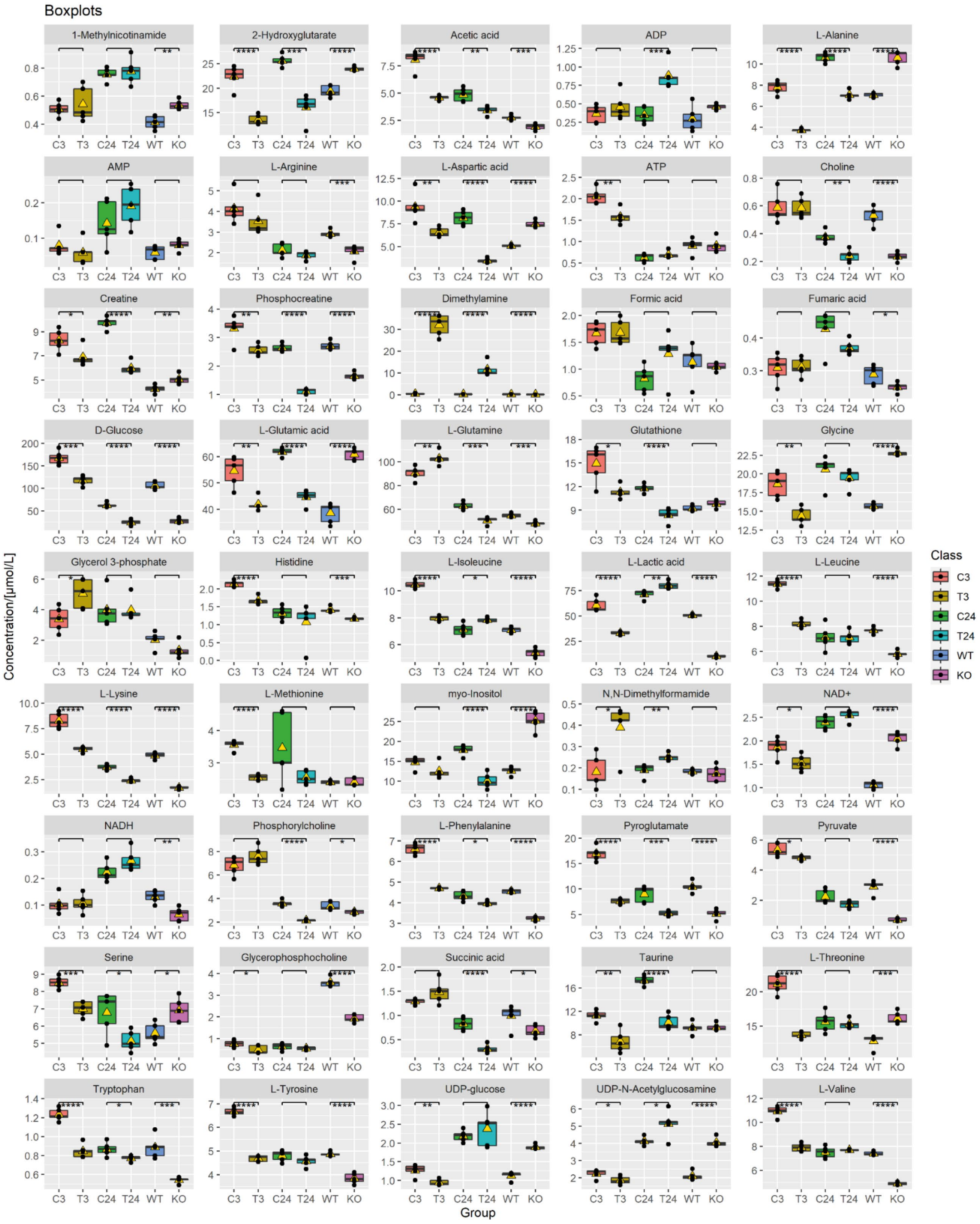

Supplementary Figure 17 (related to Figure 5): Comparison of metabolite concentrations measured by NMR. Boxplots of metabolites identified in the different trial groups, using normalized data. C3/T3: cells treated for 3 h with the DMSO solvent control (0.033% DMSO) or with 33 µM CP1; C24/T24: cells treated for 24 h with the DMSO solvent control (0.1% DMSO) or 100 µM CP1; KO/WT: PGP-KO (clone C2) and PGP-WT (clone D5) cells treated

for 24 h with the DMSO solvent control (0.1% DMSO). The black bars show the median of a distribution, while the yellow triangles show the average. A box is drawn from the 25th to the 75th percentile. Statistical testing was performed with two-sided *t*-tests for the comparisons T3-C3, T24-C24 and KO-WT. Statistically significant differences are indicated by asterisks, with * representing $p < 0.05$; **, $p<0.01$; ***, $p<0.001$; and ****, $p<0.0001$, respectively. All data are from $n=5$ biologically independent experiments. Source data including exact *p*-values are available as Source Data File, and raw datasets are available [1].

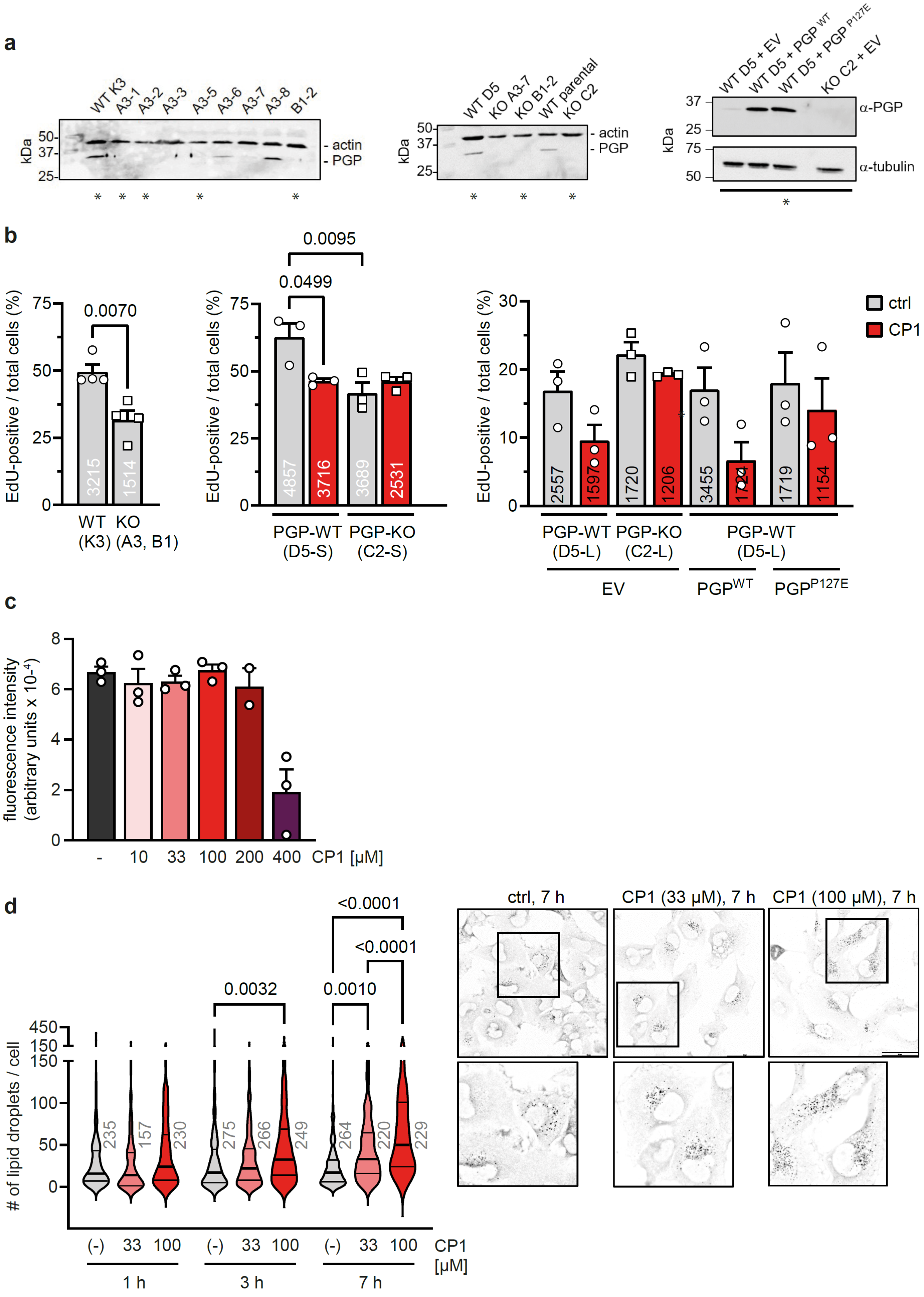

a
WT K3
A3-1
A3-2
A3-3
A3-5
A3-6
A3-7
A3-8
B1-2
kDa
50
37
25
- actin
- PGP
WT D5
KO A3-7
KO B1-2
WT parental
KO C2
WT D5 + EV
WT D5 + PGP WT
WT D5 + PGP P127E
KO C2 + EV
α-PGP
α-tubulin
b
EdU-positive / total cells (%)
0.0070
WT KO
(K3) (A3, B1)
0.0095
0.0499
PGP-WT PGP-KO
(D5-S) (C2-S)
PGP-WT PGP-KO
(D5-L) (C2-L)
PGP-WT
(D5-L)
EV
PGPWT
PGPP127E
ctrl
CP1
c
fluorescence intensity
(arbitrary units x 10-4)
CP1 [µM]
d
# of lipid droplets / cell
<0.0001
<0.0001
0.0032
0.0010
1 h
3 h
7 h
CP1
[µM]
ctrl, 7 h
CP1 (33 µM), 7 h
CP1 (100 µM), 7 h

Supplementary Figure 18 (related to Figure 5): Characterization of PGP-WT and PGP-KO HT1080 cell lines. (a) Western blots of PGP-WT and PGP-KO clones. Stars: cell lines used in further assays. Uncropped blots in Source Data. (b) Analysis of cell proliferation. Left and middle, proliferation of PGP-WT or PGP-KO single cell clones derived from cells transfected with empty vector (K3, D5), single PGP-directed gRNAs (A3, B1), or five pooled PGP gRNAs (C2). D5 and C2-clones were analyzed at passages 5-8 (short-term culture, S). Right, proliferation of PGP-WT D5 and PGP-KO C2 clones after >15 passages (long-term culture, L). Cells were transfected with empty vector (EV), PGP-WT or PGP-P127E, and treated with solvent control (ctrl, 0.1% DMSO) or CP1 (100 μM). The total number of scored cells is given in the bars. Results are mean values ± S.E.M. of *n*=4 (left) or *n*=3 (middle and right) biologically independent experiments. Statistical testing: Left, two-sided *t*-test; the *p*-value is indicated. Middle and right, ordinary one-way ANOVA and Tukey's multiple comparisons test. Multiplicity-adjusted P-values <0.05 are shown. (c) Effect of CP1 on cell viability. Cells were treated for 24 h with the indicated CP1 concentrations or 0.4% DMSO (-). Data are mean values ± S.E.M. of *n*=3 biologically independent experiments. (d) Effect of CP1 on lipid droplet (LD) formation. Left, quantification of perilipin-3-positive LDs after treatment with CP1 or the respective solvent control (-; 0.03 or 0.1% DMSO). The total number of analyzed cells in *n*=3 biologically independent experiments is indicated. Statistical testing: ordinary one-way ANOVA and Tukey's multiple comparisons test. Multiplicity-adjusted P-values <0.05 are shown. Violin plots indicate the median (middle line) and the two quartiles. Right, representative images of cells treated for 7 h with either 0.1% DMSO (ctrl), or CP1 (33 or 100 μM). Boxed areas are shown after magnification on the right. Scale bar, 50 μm. Source data are available as Source Data File.

SUPPLEMENTARY TABLES

Supplementary Table 1. PGP inhibitors (Related to Supplementary Figure 1)

	InChI Key	IC_{50} [µM]
CP1	UIRNMCRFFYWCBB-UHFFFAOYSA-N	0.4 *
	FMP-504747	0.5
CP2	YMAHKXJOPZIQNS-UHFFFAOYSA-N	0.7 *
CP3	XGPGAYNPTTXYNR-UHFFFAOYSA-N	1 *
	PGFQXGLPJUCTOI-WYMLVPIESA-N	~1
	OCLMUYZIODMVKH-UHFFFAOYSA-N	~1
	QXZNFXSPMLZFMU-UHFFFAOYSA-N	1.4
	NLLJGHICDDYOKM-UHFFFAOYSA-N	1.6
	JTDPTJCJRLGLMF-UHFFFAOYSA-N	2.1
	NIOHNDKHQHVLKA-UHFFFAOYSA-N	2.3
	VFPIHZHBUHSUJD-GZTJUZNOSA-N	2.3 *
	JCTPTAVDHVJTPQ-UHFFFAOYSA-N	3.3
	KNGOHZSJNAXLKY-SXGWCWSVSA-N	3.8
	ZTMXDMBRIAUJPC-UHFFFAOYSA-N	5.4
	WRRZZAZIGIYVLF-XGICHPGQSA-N	6.1
	IAYWMZRXAFUTJN-UHFFFAOYSA-N	10
	ZEYCTIJAPNSRIO-UHFFFAOYSA-N	11
	XMQRNZMDODXLDW-UHFFFAOYSA-N	12.9
	YSWUERTYFXXAFJ-UHFFFAOYSA-N	>20
	ZSZFUDFOPOMEET-UHFFFAOYSA-N	>20
	GYJACFGQPFXKCA-UHFFFAOYSA-N	>50
	MDBCIWYPEWNOHB-VXLYETTFSA-N	>50
	ZAFWIXYCFAXUEJ-CMDGGOBGSA-N	>50

Determination of half-maximal inhibitory constants (IC_{50}) of 23 PGP inhibitory compounds (see InChI Key for chemical substance identification) using purified murine PGP and phosphoglycolate (PG) as a substrate. Data marked with an asterisk (*) are results of n=3 independent experiments. Because of the limited quantity of most compounds available for these assays, all other data are results of n=1 determinations. ~, Approximate IC_{50} value because fitting did not converge. FMP-504747 is a compound obtained from an academic donator whose structure is undisclosed for intellectual property reasons.

Supplementary Table 2. Kinetic constants of PGP-catalyzed PG hydrolysis in the presence of CP1-3.

	CP [µM]	0	0.5	1.0	2.0	4.0
CP1	K_M [µM]	351 ± 42	772 ± 95	1091 ± 146	774 ± 160	–
	v_{max} [µmol/min/mg]	52 ± 2.7	39.9 ± 2.9	33.6 ± 3.0	17.1 ± 2.1	–
	k_{cat} [s^{-1}]	30.1 ±1.5	22.9 ± 1.7	19.3 ± 1.7	9.8 ± 1.2	–
	k_{cat}/K_M [$s^{-1}\cdot M^{-1}$]	8.6×10^4	3.0×10^4	1.8×10^4	1.3×10^4	–
CP2	K_M [µM]	406 ± 52	–	807 ± 108	1187 ± 199	1220 ± 232
	v_{max} [µmol/min/mg]	50.7 ± 2.9	–	49.7 ± 4.0	51.9 ± 5.9	36.9 ± 4.8
	k_{cat} [s^{-1}]	29.2 ± 1.7	–	28.6 ± 2.3	29.8 ± 3.4	21.2 ± 2.7
	k_{cat}/K_M [$s^{-1}\cdot M^{-1}$]	7.2×10^4	–	3.5×10^4	2.5×10^4	1.7×10^4
CP3	K_M [µM]	449 ± 51	–	799 ± 109	1093 ± 149	1324 ± 415
	v_{max} [µmol/min/mg]	51.5 ± 2.8	–	50.8 ± 4.1	54.7 ± 4.9	54.3 ± 11.9
	k_{cat} [s^{-1}]	29.6 ± 1.6	–	29.2 ± 2.4	31.4 ± 2.8	31.2 ± 6.8
	k_{cat}/K_M [$s^{-1}\cdot M^{-1}$]	6.6×10^4	–	3.6×10^4	2.9×10^4	2.4×10^4

The data are mean values ± S.D. of n=3 independent experiments. Curves were fitted and parameters and standard errors were derived using the Michaelis-Menten model in GraphPad Prism 7.04. K_M, Michaelis–Menten constant; v_{max}, maximum enzyme velocity; k_{cat}, turnover number; k_{cat}/K_M, specificity constant. The k_{cat} values were calculated from the maximum enzyme velocities using a molecular mass of 34,541 Da for PGP. DMSO concentrations were kept constant, but varied between CP1 (0.02% DMSO under all conditions) and CP2/CP3 (0.04% DMSO under all conditions). Solvent control samples contained DMSO (0.02% or 0.04%, respectively) without compound. Source data are available as Source Data File.

Supplementary Table 3: Well-populated hydrogen bonds of CP1 observed in the MD simulations.

C	CP1 acceptor	PGP donor	Fraction	Average distance [Å]	Average Angle [°]	Lifetimes Cases	Lifetimes Maximum	Lifetimes Average
AS-A	O5	SER 126 - OG	0.81	2.7	163.9	3303	241	24.49
	O2	SER 71 - OG	0.50	2.7	165.5	1037	541	48.49
	O1	SER 70 - OG	0.47	2.7	166.6	2699	1694	17.47
	O1	SER 71 - OG	0.44	2.7	164.9	1302	404	33.63
	O2	SER 70 - OG	0.40	2.7	165.8	2312	211	17.13
	O1	SER 70 - N	0.38	2.8	155.2	12530	51	3.03
	O2	SER 70 - N	0.33	2.9	159.7	12024	55	2.71
AS-B	O1	SER 70 - OG	0.66	2.7	165.7	4308	533	15.38
	O5	SER 126 - OG	0.64	2.7	163.5	2123	253	30.01
	O2	SER 71 - OG	0.61	2.7	164.6	5433	369	11.24
	O1	THR 215 - OG1	0.43	2.7	163.5	4093	204	10.47
	O1	ASN 199 - ND2	0.42	2.9	157.0	15038	52	2.80
	O2	SER 70 - N	0.33	2.9	160.7	17642	22	1.89
	O2	SER 70 - OG	0.27	2.8	161.6	6298	111	4.33
	O1	SER 70 - N	0.22	2.9	155.2	10560	49	2.05

Data are shown for H-bonds with an occurrence fraction greater than 10% over the trajectory (consisting of 100,000 frames saved in 1-ps intervals). A distance of 3.0 Å and an angle of 135° were used as cutoffs in the analysis. The lifetimes indicate the maximum and average number of consecutive frames in which the corresponding H-bond is observed within the given cutoffs; the Cases column provides the number of such blocks of consecutively maintained H-bond in the trajectory. AS-A and AS-B indicate the two active sites in the dimer. O1 and O2 are the two oxygen atoms of the buried benzoate, whereas O5 is the carbonyl oxygen of the amide at the more exposed end of CP1. Source data are available as Source Data File.

Supplementary Table 4: Biomarker analysis in PGP-KO HT1080 cells.

Metabolite	AUC	*p*-value
L-Lactic acid	1	1.27E-11
L-Valine	1	2.01E-09
Glycine	1	3.05E-09
L-Phenylalanine	1	1.09E-08
L-Lysine	1	1.82E-08
D-Glucose	1	1.18E-07
UDP-glucose	1	7.91E-07
NAD+	1	9.97E-07
L-Aspartic acid	1	1.01E-06
Glycerophosphocholine	1	1.07E-06
Phosphocreatine	1	1.67E-06
UDP-N-Acetylglucosamine	1	2.08E-06
L-Leucine	1	2.65E-06
L-Glutamic acid	1	3.31E-06
L-Alanine	1	3.40E-06
myo-Inositol	1	4.79E-06
Pyruvate	1	5.03E-06
L-Isoleucine	1	8.46E-06
L-Tyrosine	1	1.04E-05
Choline	1	1.84E-05
Dimethylamine	1	2.14E-05
2-Hydroxyglutarate	1	3.15E-05
Pyroglutamate	1	3.21E-05
Histidine	1	1.41E-04
L-Glutamine	1	1.60E-04
Tryptophan	1	2.31E-04
L-Threonine	1	5.45E-04
Acetic acid	1	7.13E-04
L-Arginine	1	7.64E-04
1-Methylnicotinamide	1	1.34E-03
Phosphorylcholine	1	1.98E-02
NADH	0.96	2.60E-03
Creatine	0.96	8.81E-03
Serine	0.92	1.31E-02
Fumaric acid	0.88	2.94E-02
AMP	0.88	*9.24E-02*
Succinic acid	0.84	3.11E-02

PGP-WT (clone D5-L) and PGP-KO (clone C2-L) HT1080 cells generated by CRISPR/Cas9 engineering were subjected to untargeted NMR metabolomic analyses (n=5 biologically independent experiments). Classic univariate ROC curve analysis was used to calculate AUC values, and statistical testing was performed with two-sided t-tests. Significantly different metabolites that represent potential biomarkers (area under the curve/AUC values ≥0.8, *p*-

values <0.05) are listed. Metabolites that are not significantly impacted according to this analysis are given in italics. Source data are available as Source Data File, and raw datasets are available [1].

Supplementary Table 5: Biomarker analysis in CP1-treated HT1080 cells.

Metabolite	AUC	*p*-value
Phosphocreatine	1	3.40E-08
L-Alanine	1	9.66E-07
L-Aspartic acid	1	1.24E-06
Phosphorylcholine	1	2.02E-06
L-Glutamic acid	1	2.12E-06
Creatine	1	2.40E-06
D-Glucose	1	4.96E-06
Taurine	1	5.77E-06
L-Lysine	1	1.00E-05
Dimethylamine	1	5.19E-05
myo-Inositol	1	7.01E-05
Glutathione	1	7.78E-05
Succinic acid	1	8.12E-05
L-Glutamine	1	1.81E-04
ADP	1	5.19E-04
Pyroglutamate	1	9.51E-04
Choline	1	1.18E-03
Acetic acid	1	2.01E-03
N,N-Dimethylformamide	1	4.41E-03
L-Lactic acid	1	8.14E-03
2-Hydroxyglutarate	1	1.26E-04
L-Phenylalanine	0.96	1.29E-02
L-Isoleucine	0.92	1.02E-02
Tryptophan	0.88	2.72E-02
Serine	0.88	2.90E-02
UDP-N-Acetylglucosamine	0.84	2.49E-02

Cells were incubated with CP1 (100 µM, 24 h) or the DMSO solvent control (0.1%, 24 h), and metabolites were identified by NMR (n=5 biologically independent experiments). Classic univariate ROC curve analysis was used to calculate AUC values, and statistical testing was performed with two-sided t-tests. Significantly different metabolites that represent potential biomarkers (AUC values ≥0.8, p-values <0.05) are listed. Source data are available as Source Data File, and raw datasets are available [1].

Supplementary Table 6: Biochemical pathway analysis in PGP-KO HT1080 cells.

Pathway	*p*-value	connection
Glycolysis / Gluconeogenesis	1.29E-09	A,C,G
Aminoacyl-tRNA biosynthesis	1.54E-09	
Alanine, aspartate and glutamate metabolism	6.39E-09	A,C,G
Glyoxylate and dicarboxylate metabolism	1.41E-08	A
Lysine degradation	1.82E-08	
Biotin metabolism	1.82E-08	
Porphyrin and chlorophyll metabolism	2.81E-08	A
Valine, leucine and isoleucine degradation	3.10E-08	C
Pantothenate and CoA biosynthesis	3.48E-08	A
Arginine and proline metabolism	4.46E-08	A
Glycine, serine and threonine metabolism	5.50E-08	A
Histidine metabolism	8.13E-08	A
D-Glutamine and D-glutamate metabolism	1.12E-07	A,C
Nitrogen metabolism	1.12E-07	A
Valine, leucine and isoleucine biosynthesis	1.70E-07	
Galactose metabolism	2.34E-07	
Ascorbate and aldarate metabolism	2.34E-07	C
Phenylalanine, tyrosine and tryptophan biosynthesis	3.46E-07	
Phenylalanine metabolism	3.46E-07	
Pyruvate metabolism	4.11E-07	G
Amino sugar and nucleotide sugar metabolism	4.35E-07	A
Pentose and glucuronate interconversions	7.91E-07	
Starch and sucrose metabolism	7.91E-07	G
Ether lipid metabolism	1.07E-06	
Tyrosine metabolism	1.33E-06	C
Nicotinate and nicotinamide metabolism	2.09E-06	A
beta-Alanine metabolism	3.39E-06	A
Selenocompound metabolism	3.40E-06	A
Glycerophospholipid metabolism	3.63E-06	
Glutathione metabolism	4.02E-06	A
Arginine biosynthesis	4.77E-06	C
Inositol phosphate metabolism	4.79E-06	
Phosphatidylinositol signaling system	4.79E-06	
Ubiquinone and other terpenoid-quinone biosynthesis	1.04E-05	
Citrate cycle (TCA cycle)	3.72E-05	A,C,G
Butanoate metabolism	4.62E-05	A
Primary bile acid biosynthesis	8.94E-05	
Pyrimidine metabolism	1.60E-04	A
Cysteine and methionine metabolism	1.77E-04	

Metabolites that were significantly altered in PGP-KO versus PGP-WT cells (see Supplementary Table 4) were subjected to biochemical pathways analysis using the KEGG database. Classic univariate ROC curve analysis was used to calculated AUC values, and pathway enrichment analysis was performed using MetaboAnalyst. Pathway *p*-values were

assigned according to the number of altered metabolites and the separation of the respective metabolite concentrations in the control and trial groups, employing the MetaboAnalyst options global test and relative-betweenness centrality in combination with the KEGG Homo sapiens database. The right column (connection) indicates pathways directly connected to the alanine, aspartate and glutamate pathway (A), the citric acid cycle (C), and/or to glycolysis (G). Source data are available as Source Data File, and raw datasets are available [1].

Supplementary Table 7: Biochemical pathway analysis of CP1-treated HT1080 cells.

Pathway	*p*-value	connection
Alanine, aspartate and glutamate metabolism	2.65E-07	A,C,G
D-Glutamine and D-glutamate metabolism	3.26E-07	A,C
Nitrogen metabolism	3.26E-07	A
Arginine biosynthesis	7.01E-07	C
Selenocompound metabolism	9.66E-07	A
Aminoacyl-tRNA biosynthesis	2.04E-06	
Glycolysis / Gluconeogenesis	3.29E-06	A,C,G
Histidine metabolism	3.55E-06	A
Butanoate metabolism	4.74E-06	A
Taurine and hypotaurine metabolism	5.77E-06	
Arginine and proline metabolism	7.32E-06	A
Lysine degradation	1.00E-05	
Biotin metabolism	1.00E-05	
Glyoxylate and dicarboxylate metabolism	1.46E-05	A
Pantothenate and CoA biosynthesis	5.34E-05	A
Glutathione metabolism	6.19E-05	A
Inositol phosphate metabolism	7.01E-05	
Phosphatidylinositol signaling system	7.01E-05	
Propanoate metabolism	8.12E-05	G
Glycerophospholipid metabolism	1.77E-04	
Pyrimidine metabolism	1.81E-04	A
Galactose metabolism	3.45E-04	
Ascorbate and aldarate metabolism	3.45E-04	C
Purine metabolism	3.76E-04	A
Glycine, serine and threonine metabolism	5.11E-04	A
Citrate cycle (TCA cycle)	8.91E-04	A,C,G
Nicotinate and nicotinamide metabolism	1.15E-03	
Primary bile acid biosynthesis	1.42E-03	
Pyruvate metabolism	1.45E-03	G
beta-Alanine metabolism	1.46E-03	A
Porphyrin and chlorophyll metabolism	1.56E-03	A
Phenylalanine, tyrosine and tryptophan biosynthesis	1.78E-02	
Phenylalanine metabolism	1.78E-02	
Cysteine and methionine metabolism	2.15E-02	
Tryptophan metabolism	2.72E-02	
Tyrosine metabolism	2.89E-02	C

Metabolites that were significantly altered in CP1- versus mock-inhibited cells (see Supplementary Table 5) were subjected to biochemical pathways analysis using the KEGG database. Classic univariate ROC curve analysis was used to calculated AUC values, and pathway enrichment analysis was performed using MetaboAnalyst. Pathway *p*-values were assigned according to the number of altered metabolites and the separation of the respective

metabolite concentrations in the control and trial groups, employing the MetaboAnalyst options global test and relative-betweenness centrality in combination with the KEGG Homo sapiens database. The right column (connection) indicates pathways directly connected to the alanine, aspartate and glutamate pathway (A), the citric acid cycle (C), and/or to glycolysis (G). Source data are available as Source Data File, and raw datasets are available [1].

SUPPLEMENTARY NOTES

SUPPLEMENTARY NOTE 1

PGP and metabolite repair processes in glycolysis

PGP is the only mammalian enzyme that has been described to act on 2-phosphoglycolate, L-2-phospholactate and 4-phosphoerythronate, and there are no known functional overlaps with other metabolite repair processes in the context of glycolysis [2,3]. Specifically, the nine other metabolite repair enzymes in glycolysis and their substrates are: (1) fructosamine 3-kinase: ε-fructoselysine; (2) magnesium-dependent phosphatase-1: protein-bound ε-fructoselysine-6-phosphate; (3) glucose-6-phosphatase catalytic subunit 3 in cooperation with glucose-6-phosphate translocase: 1,5-anhydroglucitol-6-phosphate; (4) NAD(P)HX dehydratase: *S*- and *R*- NAD(P)HX; (5) NAD(P)HX epimerase: *S*- and *R*- NAD(P)HX; (6) acylphosphatase-1 or phosphoglycerate kinase: 1,4-bisphospho-erythronate; (7) L-2-hydroxyglutarate dehydrogenase: L-2-hydroxyglutarate; (8) glutathione-dependent glyoxalase-1: methylglyoxal; (9) glyoxalase-2: methylglyoxal [2].

As a phosphatase, PGP may target the phosphorylated metabolites formed in (2), (3) and (6). This possibility seems nevertheless unlikely for the following reasons:

To (2): Magnesium-dependent phosphatase-1 (MDP-1) is a C0-capped member of the HAD phosphatase family that dephosphorylates protein-bound fructosamine 6-phosphates, without any detectable activity on free fructosamine 6-phosphates [4]. Given that PGP is a C2-capped HAD phosphatase, it is structurally unlikely that the physiological substrate spectra of the protein-directed MDP-1 and the small metabolite-directed PGP overlap [5].

To (3): 1,5-Anhydroglucitol-6-phosphate is a side product, and at the same time a strong inhibitor of hexokinases. This non-canonical metabolite is produced in the cytosol, and eliminated by glucose-6-phosphate translocase-mediated transport into the lumen of the

endoplasmic reticulum, where it is dephosphorylated by the so-called glucose-6-phosphatase catalytic subunit 3, a transmembrane protein with only negligible glucose-6-phosphatase activity. Patients or mice with genetic deficiencies in glucose-6-phosphate translocase or in glucose-6-phosphatase catalytic subunit 3 accumulate 1,5-anhydroglucitol-6-phosphate and present with severe neutropenia and susceptibility to infections [6], indicating that PGP cannot compensate for a loss of function in this metabolite repair system. Hence, it seems improbable that there is a functional overlap, which might also be due to the fact that PGP is a cytosolic enzyme, whereas 1,5-anhydroglucitol-6-phosphate detoxification takes place in the endoplasmic reticulum.

To (6): 1,4-Bisphospho-erythronate is adventitiously formed from erythrose 4-phosphate in a glyceraldehyde 3-phosphate dehydrogenase (GAPDH)-catalyzed side reaction. 1,4-Bisphospho-erythronate is a reaction intermediate, which is subsequently dephosphorylated to 4-phospho-erythronate (a PGP substrate) by acylphosphatase-1 or phosphoglycerate kinase. *In vitro* assays with erythrose 4-phosphate, GAPDH and acylphosphatase-1 in the absence or presence of PGP have shown that acylphosphatase-1 stimulates the formation of 4-phosphoerythronate in the absence of PGP, and the formation of erythronate in the presence of PGP [3]. Hence, acylphosphatase-1 does not dephosphorylate 4-phosphoerythronate, and PGP does not dephosphorylate 1,4-bisphospho-erythronate. The activities of these two phosphatases therefore do not functionally overlap.

SUPPLEMENTARY NOTE 2

Intracellular concentrations of CP1

Using LC-MS experiments in HT1080 cells (see Methods), intracellular CP1 concentrations were estimated to be 0.126 ± 0.009 μM or 0.234 ± 0.005 μM CP1 upon incubation with 33 μM

CP1 for 3 h, or 100 µM CP1 for 7 h, respectively (data are mean values ± S.E.M. of n=3 biologically independent experiments).

SUPPLEMENTARY NOTE 3

Adaptation of HT1080 cell proliferation to PGP deficiency

PGP-KO C2 cells proliferated less than PGP-WT D5 cells, yet this phenotype was reproducibly lost when cells were cultured for >15 passages (see Supplementary Figure 18). To investigate the molecular basis of this adaptation, we attempted to measure 2-phospho-L-lacate and 4-phosphoerythonate levels in PGP-KO C2 compared to PGP-WT D5 cells cultured for shorter (passage 5; P5) or longer periods (passage 15; P15). Because these metabolites were not visible by NMR, we conducted mass spectrometric analyses. While 4-phosphoerythonate could be unequivocally identified, we were unable to separate 2-phospho-L-lactate from isomeric metabolites such as glycerone- or glyceral phosphate. Still, we found that the levels of 4-phosphoerythonate were strongly elevated in PGP-KO C2 cells compared to PGP-WT D5 cells at both lower and higher passages (normalized peak areas in PGP-WT P5: 889 ± 469; PGP-KO P5: 40,612 ± 3331; PGP-WT P15: 1790 ± 688; PGP-KO P15: 24,901 ± 2930 arbitrary units. Data are mean values ± S.E.M. of n=5 biologically independent experiments). These results show that at P5, 4-phosphoerythonate levels were ~46-fold higher in PGP-KO compared to PGP-WT cells, whereas at P15, they were only ~14-fold higher in PGP-deficient cells. These results reveal how HT1080 cells can partially compensate for PGP loss over time.

SUPPLEMENTARY DISCUSSION

Intracellular concentrations of CP1

Mass spectrometry was performed to determine the intracellular CP1 concentrations of HT1080 cells treated with 33 or 100 µM CP1 (see Supplementary Notes). The estimated CP1 concentrations are slightly below, but in the range of PGP IC_{50} levels determined *in vitro* (CP1 IC_{50} is 0.39 µM for human recombinant PGP; see Figure 1A). CP1 concentrations in cells may nevertheless reach PGP IC_{50} levels for the following reasons: (1) It is unknown if the potency of CP1 for recombinant PGP *in vitro* is identical to its potency towards endogenously expressed PGP. (2) The effective distribution volume for small molecules in the cytoplasm is lower than the total cell volume. (3) The average concentration of a compound in a cell lysate is unlikely to reflect its local subcellular concentrations. (4) Importantly, intracellular CP1 concentrations are not linearly correlated with the extracellularly applied CP1 concentrations. Based on the results obtained after treatment with 33 µM CP1, the expected intracellular CP1 concentration after incubation with 100 µM CP1 should be 0.382 µM, not 0.234 µM. Hence, we detected 39% less CP1 than expected. These data indicate that CP1 is intracellularly metabolized or degraded. Because only intact CP1 could be quantified (nothing is known about the intracellular metabolism or degradation products of this compound), we conclude that the intracellularly effective CP1 concentrations are likely underestimated.

These results also explain why relatively large CP1 concentrations are required in PGP-dependent cell proliferation and lipid droplet accumulation assays (see Figure 5). In addition to CP1 metabolism/degradation, it is also possible that the cellular uptake of CP1 is limiting, and/or that CP1 is rapidly exported.

CP2, CP3 and structural analogs

Although the present study focuses on CP1 as a prototypical PGP inhibitor, CP2 and its analogs such as 2B, as well as CP3 have similar PGP inhibitory activities and binding affinities (CP3). Although we have not yet conducted experiments beyond the ones reported in this study, CP2 and CP3 or their structural analogs are of interest and could be investigated further.

SUPPLEMENTARY REFERENCES

1. Alwahsh, M., Hollmann, R., Hergenröder, R. & Gohla, A. . NMR raw data to article: Glycolytic flux control by drugging phosphoglycolate phosphatase. . 12.10.2022 edn (Figshare, 2022).
2. Bommer, G.T., Van Schaftingen, E. & Veiga-da-Cunha, M. Metabolite Repair Enzymes Control Metabolic Damage in Glycolysis. *Trends Biochem Sci* **45**, 228-243 (2020).
3. Collard, F. et al. A conserved phosphatase destroys toxic glycolytic side products in mammals and yeast. *Nat Chem Biol* **12**, 601-7 (2016).
4. Fortpied, J., Maliekal, P., Vertommen, D. & Van Schaftingen, E. Magnesium-dependent phosphatase-1 is a protein-fructosamine-6-phosphatase potentially involved in glycation repair. *J Biol Chem* **281**, 18378-85 (2006).
5. Gohla, A. Do metabolic HAD phosphatases moonlight as protein phosphatases? *Biochim Biophys Acta Mol Cell Res* **1866**, 153-166 (2019).
6. Veiga-da-Cunha, M. et al. Failure to eliminate a phosphorylated glucose analog leads to neutropenia in patients with G6PT and G6PC3 deficiency. *Proc Natl Acad Sci U S A* **116**, 1241-1250 (2019).
