# Supplementary material for: Glycolytic flux control by drugging phosphoglycolate phosphatase
Source: Nat Commun. 2022 Nov 11;13:6845. doi: 10.1038/s41467-022-34228-2 (PMC9652372; doi:10.1038/s41467-022-34228-2)
Supplement: Supplementary file 4 — Description of Additional Supplementary files [file 41467_2022_34228_MOESM4_ESM.pdf]

Description of Additional Supplementary Files

File name: Supplementary Movie 1 (related to Supplementary Figure 13).
Description: Movie illustrating the movements along the first principal component (PC1) for apo-PGP.

File name: Supplementary Movie 2 (related to Supplementary Figure 13).
Description: Movie illustrating the movements along the second principal component (PC2) for apo-PGP.

File name: Supplementary Movie 3 (related to Supplementary Figure 13).
Description: Movie illustrating the movements along the first principal component (PC1) for CP1-PGP.

File name: Supplementary Movie 4 (related to Supplementary Figure 13).
Description: Movie illustrating the movements along the second principal component (PC2) for CP1-PGP.
